# Notes on Medical Matters and Medical Men in Paris

**Published:** 1847-08

**Authors:** David W. Yandell

**Affiliations:** Louisville, Ky.; Paris


**Art. II.**—*Notes on Medical Matters and Medical Men in Paris.* By David W. Yandell, M.D., of Louisville, Ky.

It is my intention, at no very distant day, in a letter on the Physicians and Surgeons of Paris, to go somewhat into detail respecting a few of the most prominent of these, while at the same time I shall pass rapidly in review some of their peculiar doctrines. In this way, I fancy, more successfully than in any other, I shall be able to convey a correct idea, which, I must say, does not exist in the mind of every American physician, concerning the medical luminaries of the French capital. This, however, at another time; in the meanwhile I give you a report of one of the lectures of Prof. Bouillaud, one of the greatest of French physicians. Since it appears to me to be superior to anything I have yet seen on the subject—*Meningitis*—I propose to communicate it in full.

The disease, he remarked, the study of which we enter upon this morning, has been successively termed ataxic fever, brain fever, and arachnitis, but the name which seems to us best to express the characters of the affection is meningitis. True, it does sometimes occur that the arachnoid alone is diseased, its cavity containing serum or pus, or patches of pseudo-membrane of greater or less extent; but independently of these cases being extremely rare, it appears in the greater part of those that have been published, that the inflammation was not confined to the serous membrane. Moreover, the principal, at least, if not the exclusive seat of the inflammation is the cellular tissue underneath the arachnoid, that is to say, the pia mater. The dura mater participates in the inflammatory action only because of its contiguity and of a persistence of that action, it being only after a chronic inflammation that we discover the membrane thickened, degenerated, or covered by cartilaginous or osseous deposits. The bones of the cranium themselves may undergo alterations under such circumstances.

Meningitis may be general or partial, it being understood that we confine ourselves to cranial meningitis. In this we include that which affects the periphery of the hemispheres or their superior parts; that which affects the base of the brain, and finally, the meningitis which is seated in the ventricles alone. The inflammation may equally affect the periphery of the organ alone or the parietal surface of the meninges. M. Foville has particularly described the latter, which I may remark very rarely exists independently of inflammation of the other portions of the meninges. Its most frequent causes are external violence, as wounds and fractures of the back of the cranium.

*Anatomical characters.*—These do not differ essentially from those of inflammation in other parts of the body, excepting certain peculiarities which are offered by reason of the structure and properties peculiar to the affected tissues.

The first or acute period, betrays itself by a redness which may be immediately carried to so high a degree as to resemble a true sanguine effusion in the substance and on the surface of the tissues; but it is ordinarily more manifest beneath the arachnoid, in the folds of the pia mater. Most frequently, however, both the arachnoid and pia mater are only covered by a simple arborescence, or a more or less abundantly dotted appearance, while they are at the sametime turgescent and more or less tumefied. Nothing more is required than heat and pain to characterize inflammation such as we have described in a pathological point of view, but these are merely physiological phenomena which it is impossible to demonstrate after the death of the patient.

In this state of things the tissues actually lose their cohesion to such a degree that the pia mater and arachnoid, the choroid plexuses, and the membranes of the ventricles become extremely friable, being easily torn under the finger. This is what is designated under the generic name of *ramollissement*, and is entirely analogous, as to its mode of formation, to the *ramollissement* of the brain and parenchymatous organs.

At this period these membranes are sometimes covered by granulations, which, however, are seen more especially in chronic meningitis, or rather that particular form of the affection called tubercular meningitis. The surfaces of the arachnoid and of the ventricles are moistened and lubricated in the normal state, you are aware, by a serous liquid; but there does not exist, strictly speaking, any serum in their cavities, and when it is found it has been formed during the last moments of life. A secretion should be considered as the result of an inflammation only where the chemical properties of the fluid, serum for instance, are changed. Now, in the majority of cases, we find sometimes the arachnoidian cavity, sometimes that of the ventricles, distended by a considerable quantity of serum, still transparent, though somewhat less so than in the natural state, and a little more thick. Such is the true transition from the moment of the inflammatory state. It is the active dropsies which constitute a state of protophlogosis, if we may so speak, and which only require another degree to be added in order to become a true inflammation. The symptoms of this protophlogosis are in other respects identical with those of inflammation properly so called, which do not differ from the first except in the character of the secretion. Thus, in the one, we see a serous liquid, perhaps a little thickened, filling the ventricular and arachnoid cavities; whilst in the second this liquid is thicker, viscous, opaque, most frequently infiltrated beneath the arachnoid, in the meshes of the pia mater, when it presents itself under the demiconcrete form of gelatin. The arachnoid and the membranes of the ventricles seem to imbibe this liquid, and to owe to this imbibition their thickening and expansion, at the same time their cavities are filled with the liquid, in which case it generally happens that it eventually divides at the surface and cavity of the membranes into two parts, the one fluid and very limpid, the other flocculent, amorphous, and pulpy. Thus, although there is no great difference as to the mode of formation of these two degrees of the same disease, they are extremely distinct if we view only their anatomical alterations. In fact the one, active dropsy, (protophlogosis) remains stationary and always furnishes an abundant liquid which does not vary, unless a new action, a greater intensity, is superadded which converts it into a true inflammation. This, on the contrary, furnishes a thick milk-like serum, which in the greater number of instances becomes readily converted into pseudo-membranes more or less dense; and these usually become so far organized as to be furrowed with reddish striæ, indicating the formation of new vessels. If the disease, instead of terminating abruptly in death, as most frequently it does, continues beyond three or five weeks, for example, the false membranes are perfectly organized, and make a sort of integral portion of the membranes of the arachnoid and ventricles; the inflammatory movement loses its intensity, the secreted liquid is produced less turbid, less thick, and more limpid; the effusion augments, and to the inflammotory succeeds a simple hypertrophic action, in so much that the secretion, now more abundant, retains all the characters that it had in the first instance.

M. Foville in his article on meningitis has spoken of false membranes lining all the cavity of the arachnoid, and which almost constituted a new cavity. We ourselves examined a very remarkable case of this kind last year. Leaving aside false membranes, it is probable that many cases of hydrocephalus have no other origin than this new cavity. We observe then, as after a dropsy, milky patches very thin and adherent to the internal surface of the serous membranes.

But these things do not always terminate in this way, and to the injection and thickening of the membranes, to the secretion which occurs in the first moments of meningitis, succeeds soon a more or less abundant suppuration. In certain cases we have found the pia mater steeped in a very consistent phlegmonous pus; occasionally both the internal surface of the arachnoid and the ventricles, according as either one or the other of these parts have been the seat of the inflammation, are thus covered; and it is also frequently found beneath the arachnoid in the meshes of the pia mater, when its position may be exactly ascertained by pressing lightly upon the serous membranes, which will cause it to change its postion beneath its envelop, the arachnoid.

Besides these anatomical lesions, there often supervene granulations more or less perfectly organized, true tubercles which occupy either the surface of the arachnoid or the substance of the pia mater itself, though these do not occur in all cases, but particularly, I may say almost exclusively, in lymphatic individuals who present also in the majority of instances tubercular peritonitis, as well as those pericardites and tuberculous pleurisies, that you will meet so often in practice.

We do not partake of the opinions of those writers, and they are many, who contend that these tubercles existed anterior to the inflammation of the meninges, which, in the end, they produce; we think, on the contrary, that they are constantly preceded by the inflammation which produces them, and when once developed, if this inflammation subsides, or is arrested in its march by any circumstance whatever, they (the tubercles) may exist for years without giving rise to any inflammation or producing any further anatomical alterations, just in the same way, for example, that granulations supervene on the valves of the heart in consequence of a violent and neglected endocarditis. Now what is the nature of these granulations, of these tubercles, that authors have but too superficially examined, and that they agree, without knowing why, to regard as a *sui generis* affection, the true generative cause of which they have never seemed to wish to recognize? This is a question which, in a pathological point of view, is as important as it is vast, since it naturally leads us to speak not only of tubercles of the meninges, but of tubercles in general. And the digression which I am about to make will not, I trust, be considered out of place. We do not hesitate to express ourselves on this subject in the most positive manner. Let those who regard tubercles as something peculiar in their nature, or who do not believe that there exists any relation between inflammation and their production, be offended at our opinion; for us this opinion is not the less demonstrated, and it will certainly be so for all those who observe well. And first we lay down as a fact, that inflammation alone is the producing cause of tuberculous matter, in a word, of tubercles. Let us now add, that it is inflammation of the lymphatic system, of the lymphatic vessels, which determines the secretion, *sui generis*, it is true, which constitutes tuberculous lesions properly so called. The first support of our proposition is furnished by the fact, recognized and admitted on every hand, that of all individuals those among whom the lymphatic system predominates are the most subject to tubercles. Now if it is said that tubercles are a special disease developed idiopathically and primitively, and we are given as proof the transmission from father to son, in a hereditary manner, of the tuberculous diathesis; we reply without fear of being contradicted by sound observation, that it is by no means tubercles that are inherited, but rather the temperament predisposing to the tuberculous affection; the predisposing cause being, *par excellence*, the lymphatic temperament. Every one knows with how few exceptions all temperaments are hereditary. It is the same with tubercles as with inflammatory diseases properly so called, or the different organic diseases which are occasioned by them. These inflammatory diseases belong to sanguineous temperaments every one will allow; and certainly no one would be found bold enough to assert that pneumonia, for example, is a hereditary affection, though the temperament which predisposes to it is hereditary. But before the connexion which existed between chronic organic affections and the acute inflammations which precede them, was known positively as it is to-day, before it was known that these chronic organic maladies, like the anatomical lesions which charaterized them, were but the more or less perfectly transformed and organized remains of an acute anterior inflammation; before this epoch,we say,what was the opinion entertained of these very alterations, and only to speak of a single organ, the heart, what was said of the cause of its diseases? Aneurism was regarded as one of the pretended hereditary affections precisely as are tubercles. Yet heirship was not esteemed the sole and only cause capable of engendering them, and the supporters of the doctrines admitted, perhaps compulsively, accidental causes in the same way that those who believe in the hereditary transmission of tubercles are also constrained, in certain cases, to admit that this mysterious germ may be developed spontaneously and accidentally, without knowing how or under what influences; but in the majority of instances under the influence of the lymphatic temperament.

Thus then, an individual with a more or less predominant lymphatic temperament is predisposed to tubercles, and is unfortunately very often attacked without having had tuberculous progenitors. Such in our opinion, is the predisposing cause, *par excellence*, nay, we would say almost the only one, of tuberculous disease.

We draw another support of this opinion from a large class of surgical diseases. Do we not daily see excoriations, wounds, contusions, in a word, traumatic lesions of every kind, followed by a more or less intense inflammation of the lymphatics comprised in the wound or its neighborhood, and by engorgement of the ganglia to which the intersected vessels extend? And when the first period of this adenitis has passed, if it has not been properly treated, the ganglia tuberculize and are transformed into masses of the best characterized tuberculous matter. We remember to have had a most striking example of this transformation in a patient who succumbed to a pleuro-pneumonia and an abundant albuminaria, in whom all the ganglia of the parotid and submaxillary regions formed a tumor almost as resisting as if it had been osseous, while its volume was nearly quadruple the natural size of the ganglia. Being incised it was found to consist entirely of yellowish masses of a soft granular matter, resembling, save the color, a mixture of plaster previously dissolved in water, and commencing to solidify, studded with large granulations. Some points were completely softened, and yielded under pressure a white yellowish pus, offering the most distinct characters of soft tubercle or, in other words, of tuberculous pus. All the mesenteric ganglia indifferently presented the same degeneration.

If we see then in these external diseases tubercles developed, if we see them engendered by an incontestable inflammation of the ganglia and lymphatic vessels; if, finally, we see this tuberculization arise and progress step by step under our eyes, in the lymphatics of the external organs, we ask if we are not forced by the most pressing analogy and the most rational induction to admit, that it is not produced otherwise in the internal organs? Would that all the inductions that have been and will be made in medicine were as well founded as this; the science would progress much more rapidly; true principles would be much more numerous, and false principles fewer than they are, and an immense number of facts, isolated because they have been imperfectly studied, and badly studied because no one has dared lift the mysterious veil which envelops them, or rather which necessarily enveloped them when science was still in its infancy, would now be assimilated, classified, grouped, united and transformed into general laws. The great error of many persons consists in believing, by an inconceivable tendency to see on every hand mysteries and new prodigies, that nature acts in a thousand different ways, whilst, on the contrary, it is a fact which is confirmed by daily observation, and which you should never lose sight of, that nothing is more constant, more unerringly identical than the march and effects of a disease in the same tissue, in the same organ, or the same system of organs, if the disease, inflammation, for example, be left under the same natural influences to the hands of nature.

Finally, what are tubercles, and what the relation of cause and effect between tubercles and inflammation of the lymphatic vessels? Tuberculous matter is to inflammation of the lymphatic system what false membrane is to inflammation of the sanguineous system. Thus whenever an inflammation becomes general in the sanguine system it produces a peculiar *secretion*, an exudation from the internal membrane of the vessels. It is swept away by the blood in proportion as it is produced, either in part or wholly; and fibrin being added the blood then becomes more glutinous, coagulable, and plastic, as is shown by the buffy coat, which is a true organic germ, a veritable elementary tissue, only requiring to be grafted upon a living organ in order to become endowed with a life of its own.

The same thing, moreover, occurs in a large number of instances when poured out on the surface of the serous membranes, for example, or into the interstices of the cellular tissues by the capillaries, when it assumes the form of false membranes; and then if these are not absorbed by the force of nature alone, or by nature aided by therapeutic agents, they soon commence to become organized in a definite manner, to become vascular and to be transformed into true serous and fibrous membranes, which may themselves be changed at a later period, by virtue of a power of nutrition possessed by them, the mechanism of which escapes us, into cartilaginous, osseous, or osseo-calcareous matter.

You all know that in another degree of inflammation of the circulating system, and especially in local inflammations of this system, obliteration of the vessels is the result, both by the plastic matter exuded and the coagulation of the blood, and the clots are reduced in a short time to true fibrin. These are more or less adherent to the vascular parietes, and are themselves organized in a great number of instances, and as we have before remarked while speaking of false membranes, they occasionally finish by being transformed, and in certain cases by the continuation or the increase of the inflammatory action, and especially in consequence of a phlebitis, they become, like the inflamed cellular tissue, the seat of very frequent suppuration.

Finally, when inflammation reaches the suppurative stage, an exudation, a purulent secretion, is immediately established in the capillary vessels, and we have then primitively these purulent effusions in the serous cavities which are not organizable, but true foreign bodies, which nature constantly endeavors to get rid of by means of absorption or otherwise. All that we have said relative to the products of inflammation of the circulatory system, may be repeated of the products of inflammation of the lymphatic system. Thus a coagulable liquid lymph circulates in the lymphatic vessels, as blood circulates in the blood vessels. Like the latter, the lymphatics, during the first degree of inflammation, especially when adhesive, exude from their internal surfaces a matter organizable like pseudo-membrane, and differing only very slightly from this in its nature, being tuberculous, the germ of tubercle which is identical with the coagulum of lymph. At a more advanced period, this matter becoming agglomerated, may obliterate the lymphatics, become organized and transformed, or suppurate precisely as the clots of blood which are in their nature identical with the pseudo-membranes secreted by the internal surfaces of the bloodvessels, and which like it (false membrane) may become organized or suppurate.

Finally, suppuration may also be established in the lymphatics in the same manner as in the capillaries, although the product, pus, may be somewhat different.

We are able to say now what these granulations are, these tubercles that are seen on the free surface of the peritoneum, pleura, pericardium, arachnoid, and in the meshes of the serous cellular tissue, as that of the pia mater, in the course of certain inflammations, and always from preference in lymphatic individuals. They are but the product of the exudation of the capillaries which participate in the inflammation, and which are so numerous in the serous membranes and the laminated tissues which line them. They are but the fellow, the counterpart of the pseudo-membrane of the bloodvessels furnished by the lymphatic vessels. They, as it were, become organized and vascular, and engraft themselves and live in the same manner, and, playing no longer the part of foreign bodies, may sojourn many years without anything revealing their existence. Meanwhile, once organized, they have a separate existence, and live by themselves, on their own proper account.

These products of inflammation may, and too often do, become the seat of new inflammations, under whose influence they may suppurate. Such is tubercular softening in the greater number of instances. Again; these granulations may, like the pseudo-membranes, be transformed into fibrous, cartilaginous, or calcareous matter, in the course of which metamorphoses they may frequently become the cause of new inflammation in the organ which contains them, and as they were deposited by a previous inflammation, new inflammations in which these granulations themselves participate, fall into suppuration, etc.

After this long digression we return to our subject, and terminate our remarks on the anatomical characters of meningitis, concerning which we have little to add. The cerebral substance itself, in the immense majority of cases, participates in the inflammation of the meninges, either through contiguity or the continuity of the vessels. A more or less thick layer of the cortical substance is found more or less congested; sometimes it appears as if soaked in blood, while its consistence is perceptibly diminished, or in a word, it is softened. The white substance is equally congested, and presents when cut an abundantly dotted appearance, but any alteration of its cohesion is much more rarely observed, unless the inflammation had persisted sometime with considerable severity. But when the ventricular membrane is the seat of the disease the subjacent white substances, in the same manner as the gray substance covered by the pia mater, is always extremely congested, and in the majority of cases softened to a certain depth. What we have said of the participation of the cerebral substance in the inflammation of the meninges, is a fact which we could predict beforehand and without the aid of pathological anatomy; for how can we explain the delirium and the disorders of the intellect more or less profound in cerebral fever, by the existence, simply, of inflammation of the meninges?

*Signs and Symptoms.*—Meningitis, like other inflammations, gives rise to two orders of phenomena, the one being common to all inflammations, the other peculiar or proper to each. The common phenomena, you will readily understand, are denominated heat, redness, swelling, and pain. In the disease which occupies us, it is not possible, as it is in the external inflammations, to detect these phenomena in the diseased organs themselves, but they are translated to the exterior in a perfectly analogous manner. Thus in meningitis the face is animated and red, the eye brilliant and congested, pain often shows itself in a more or less intense general cephalalgia; this, however, is not always the case, for frequently it is localized in one or other portion of the head, it being usually referred by the patients to the forehead. To what is this cephalalgia to be attributed? We are far from saying that in certain cases the inflammation of the meninges is propagated to the substance of the brain, or even that the general congestion of this organ may not be the point of departure, but we are well convinced that in those cases which are most frequent—those where the pain occupies the frontal region—the cause consists either in participation in the inflammation, or the compression of some of the branches of the fifth pair of nerves which are distributed in this portion of the membranes of the brain, and which moreover are eminently sensitive. As to the tumefaction, it cannot be directly proved any more than the redness. The external heat, especially of the head and frontal region, is too evident to need mention here.

The signs proper to the affection are the following:—In the first period there exists a trouble, a disorder, an exaltation of the intellectual and moral faculties, which usually produces a violent general delirium. Besides this, infants especially are seized with convulsive movements. In certain cases the muscles are in a state of general agitation and trembling; but in other circumstances these symptoms are replaced by rigidity and very great contraction of the muscular system. Trismus is also very frequently observed, and is a doubly grave symptom, especially when it manifests itself at the onset; first, because it announces a violent and rapid inflammation, not only of the meninges but also of the base of the brain, nervous centres, etc.; and secondly, because it is impossible to administer anything to the patients. At other times, instead of trismus, the jaws are seized with a trembling and very frequent grinding of the teeth. To all these symptoms is added an exaltation, either general or special, of sensibility. This you may easily verify by pinching very slightly or merely touching the patients, who become agitated, endeavor to change their position, and utter plaintive cries.

Concerning special sensibility the pupils are strongly contracted, and when you persist in speaking to the patient he attempts to withdraw himself from the noise which fatigues him, and of which he will often complain. In the second period, to these symptoms of exaltation of the intellectual faculties, of sensibility and of motion, which characterize the first period, soon succeeds a general comatose condition. Then, if the coma is not too intense, by questioning the patient in a very high voice and with a little perseverance, he recovers momentarily from this torpor, replies in a few words, ordinarily incoherent, and immediately relapses into the same state, which if carried very far gives place to what may be termed apparent death of the functions of relation.

The face alternates with redness and pallor; the features are shrunk and contracted, especially among children; in certain subjects the face assumes an idiotic expression; the dorsal decubitus is most common, and the members are usually extended without any movement or any species of contraction; though it occasionally happens that at the beginning of this period the patients are still bent and drawn upon themselves, lying upon the side or back as during the contractions of the first period; but there is no longer any real contraction, and the limbs seem to remain in this state rather because they are incapable of extending themselves than because prevented by contraction. Cephalalgia very probably still persists in a high degree at this period and even until the fatal issue of the disease, as is evinced, among other things, by the patients, notwithstanding their coma, from time to time raising their hands mechanically towards their heads.

In certain subjects the end of the second period is characterized by subsultus tendinum, though this is not a constant phenomenon. The contraction of the pupils is a phenomenon belonging to the first period of meningitis, always coinciding with the exaltation of sensibility and general contraction, and as soon as compression of the nervous centres, or the extensive participation of the brain in the inflammation is manifested by the symptoms characteristic of the second period, somnolency, coma, etc., the pupils tend to dilate more and more. Independently of these symptoms, there are a few which should particularly fix your attention. Generally the pulse is much accelerated in the beginning of meningitis, varying from 96 to 120 and 130, this last figure being very rare; it is full and extremely resisting, except in feeble anemic patients, in whom we have found the pulse, when beating, from 96 to 100, soft, small, and but slightly resisting; it is rarely irregular and intermitting at the beginning, but its tendency is to become more and more rapid, and at the same time that it increases in frequency it becomes irregular and often intermitting. During the second period it most frequently beats from 120 to 130 times and even more, while it is small, filiform in certain cases, and not easily compressible. If in the course of a meningitis a momentary amendment occurs the pulse becomes slower, less irregular and intermittent, and gains in volume and fulness; but if to this calming of the pulse succeeds a slight acceleration, you are authorized to pronounce a fatal prognosis, and from this moment the pulse, which at the beginning had been slightly accelerated and then somewhat slower, becomes extremely frequent, beating from 130 to 140, and in the last moments even beyond that. Those of you who were here last year had opportunities of observing, in our service, some cases of meningitis which offered very well marked characters. We should not omit saying that when, during the first period of meningitis, the convulsions and the subsultus tendinum are very frequent, in some extremely rare cases it becomes exceedingly difficult to ascertain with precision the state of the pulse. The general temperature is at the same time elevated, and acompanied by moisture or sweat over the surface of the whole body. This phenomenon is almost always present during the period of reaction and exaltation, and in our opinion, those are at fault who have referred to dryness of the skin as a constant symptom in this disease. True, dryness of the skin is remarked in meningitis at the same time that the general temperature is elevated; but it most generally accompanies the second period, and is almost uniformly present in chronic or sub-inflammatory meningitis which progresses slowly. It is also proper to say, that in certain lymphatic subjects, principally children, when that form of meningitis is observed which some pathologists regard as a special disease, that is, tubercular meningitis, the skin is almost constantly very dry, but the temperature is less elevated than in the others.

The breathing, always very frequent, sometimes reaches 28, 36, and even 40 respirations per minute, is sighing principally with children, and in the course of the disease these sighs are changed into true cries, which have been called hydrocephalic cries. This is a symptom which is met with more particularly in those cases of meningitis which are accompanied by considerable effusion of serum in the cavities of the ventricles and meninges, an effusion which shows itself in subjects where tuberculous granulations are developed in the substance of the membranes. Slackening of the respiration is equally seen, but it is due in many cases to the partial or general participation of the nervous centres in the inflammation.

The first phenomena manifested on the part of the digestive organs are anorexia, intense thirst in the beginning, coated tongue, and most generally vomiting. But this latter is often a delusive symptom, which you must mistrust, for it may lead you into error if the other symptoms that we have previously mentioned are not well marked, since it is met with in a number of circumstances, and might lead you to mistake a meningitis for a commencing gastric affection, when in fact the stomach was perfectly healthy. These vomitings or efforts to vomit are observed equally in erysipelas, especially of the scalp.

Alvine evacuations do not generally take place with the same facility, but it sometimes happens that they are passed involuntarily during slight convulsions. In the beginning of the disease the patients are occasionally seized with a diarrhea, though this symptom is not among the most frequent. During the second period, or that of coma, collapse, etc., the evacuations are no longer effected, so to speak; the rectum fills, and when it becomes distended the matter escapes involuntarily under the patient.

The urine is scanty, red, acid in the beginning, now and then depositing a brick-dust sediment; and in proportion as the disease draws near a fatal termination it becomes more and more scanty, and being no longer passed voluntarily the bladder becomes fuller and fuller, and the urine only escapes drop by drop. Many patients cannot pass it even in this way; be this as it may, catheterism is urgent in both cases. The urine is then alkaline. In the last stages of the disease the respiration often becomes more and more loud, then stertorous and laborious, when it very frequently happens that the patients suddenly succumb to a true asphyxia. This occurs principally in meningitis of the base. To this, the acute form, succeeds the slow chronic form, and it is in this variety that we see those numerous examples of insanity and mental alienation, the causes and points of departure of which Foville, Colmiel, and others have so well described. Whénever physical and mental signs, such as those that characterize pneumonia, for example, cannot be obtained, and we are obliged to rely upon functional phenomena in order to support our diagnosis, which is the case in meningitis, differential diagnosis becomes of essential importance. Meningitis evinces itself, it is true, by certain local signs, as by the congested and animated state of the countenance, the engorgement and tearfulness of the eyes, the increased throbbing of the temporal arteries, etc., but as is proved by the numberless errors committed every day, these, in the majority of cases, are not sufficient. For instance, meningitis is often taken for typhoid fever of the ataxic form, and I may remark, in passing, that it is in these cases that some pretend to have seen typhoid fever without lesion of the glands of Peyer, and that some physicians attach only a slight importance to these alterations. Certainly, the ataxic state may be complicated with typhoid fever, but it is nothing more than a pure and simple complication, for these ataxic phenomena in no degree form an integral part of typhoid fever, and even if they did they are wanting in an immense number of instances, and particularly in our wards where the disease is not allowed to continue a sufficient length of time for the nervous centres to feel the influence of the general infection, whatever may be the manner in which this infection has been brought about.

The causes of meningitis are both various and different in their nature. For the sake of better studying them they have been divided into two classes, *predisposing* and *determining* causes. But, although this general division has been received, it was found necessary to make a third group which comprehends the sympathetic or reactional causes; for although these bear a certain analogy to the predisposing causes, the resemblance is not sufficiently close to allow of their being classed with them; while from those styled determining or exciting they differ very widely. Under these circumstances we feel ourselves authorized to adopt another division, by which the causes of meningitis may be comprised under four heads, and by which the subdivisions may be determined with greater precision. It is this—1st. Direct causes. 2d. Indirect causes; which may be subdivided into 1st, Direct causes: *a.* those which act materially and are common to inflammation in general; *b.* those which are immaterial, if the expression may be allowed, or, in other words, moral or intellectual, affecting specifically the nervous centres and their appendages, which are the organs in question. 2d. Indirect causes: *a.* those which are only predisposing, such as age, sex, temperament, physiological conditions, such as food, drink, etc.; *b.* those operating sympathetically or by reaction, and which comprise certain morbid states, such as typhoid fever, erysipelas of the scalp or of the face, sometimes pneumonia of the summit of the lungs, etc. Let us now enter into details—

1st. *Direct Causes.*—*a.* Those which are material and common to inflammation in general. In this, you remark, we have included all traumatic lesions, such as profound contusions of the cranium, whether occasioning fracture with depression of the skull, or only violent commotion of the brain, wounds resulting from severe operations on the scalp and the like. Under this category also is embraced a too long exposure to the rays of a hot sun. This, it is well known, may occasion erythema or erysipelas, according as the heat has been more or less intense and continued; in the same manner it may determine meningitis, provided the head be sufficiently exposed. *b.* The different organs become inflamed under the influence of causes which act upon them in a specific manner; exciting food taken in large quantities and most of the mineral poisons, produce gastritis; but exposure to cold, pneumonia, rheumatism or coryza, and by the same law, intense moral affections, such as profound grief, extreme joy, prolonged vigils, immoderate mental labor, acting powerfully on the brain, are the most usual causes of meningitis in adults, and particularly in young adults. It is necessary to remark, however, that in this case the cerebral substance generally participates either primitively or consecutively in the inflammation of its envelops.

2d. *Indirect Causes.—a.* Those which are only predisposing. The different periods of life are not liable to meningitis in the same degree. It is met with oftener before the age of ten than from ten to twenty; during the latter period it occurs more frequently than in middle life, and as old age advances it becomes more and more rare. The influence of age then upon the production of this disease is both great and easily proved; whereas in regard to sex the question is more difficult. M. M. Guersant and Foville assert, and they found their opinion on observation, that young girls are more subject to meningitis than boys, and they attribute this to the *serre têtes* commonly worn by young females in France, a species of cap occasioning a continual and considerable circular pressure upon the head.

By tables recently drawn up at the *Hôpital des Enfans*, and published in a special treatise, it appears from the article on meningitis, that girls are much less subject to this disorder than boys. To what are we to attribute these discrepancies? We cannot here enter into details, but will simply say that the question requires further study, and that, even should the opinion of Guersant and Foville prevail, the explanation which they have given cannot be the true one; for how is it possible that a constriction, which at most is not very great, and which is exerted for four, five, or six years, should determine disease at a period when, taking into account the gradual development of the head, the organs must have become in some degree habituated to such compression? It seems much more logical and just to admit the influence of constitution and of temperament,—for it is well known that young girls and even adult females are generally more lymphatico-nervous than males. If we take this side of the question, then, we should say that lymphatic and nervous temperaments are more subject than any others to meningitis. This explains to us on the one hand why it oftenest attacks infancy; and on the other, why that variety called granulous or tuberculous is almost peculiar to this epoch of life. Five children out of six are in fact more lymphatic than nervous, and we have already assumed that tubercular meningitis is the result of inflammation, and inflammation of the lymphatic system. And from these facts our opinion gathers new force and confirmation.

It is constantly observed that certain physiological conditions are real and frequent causes of meningitis. First and second dentition; the first appearance of the menses, or the sudden and complete suppression, in whatever way caused, of this periodical discharge, are each periods marked in numerous instances by the development of meningitis. These are by no means vague hypotheses to be repeated by medical authors merely because advanced by those who have preceded them, while they have had no means of assuring themselves of their truth; but facts, and what is still more weighty, groups of facts, the numerical tables given in special treatises, the exactitude of which we, ourselves, have had repeated opportunities of verifying,—these offer quite sufficient proof.

A highly stimulating diet continued for a length of time, and especially the abuse, sometimes made of spirituous liquors with the view of drowning mental suffering, are also causes, the action of which though less rapid and probably more remote, is not the less positive. You well know that excessive and protracted indulgence in spirituous liquors very commonly produces a variety of this disease, known as *meningite crapuleuse*, or delirium tremens, which is nothing more than a slow and chronic phlegmasia, of which you will always find palpable and material traces after death.

*b. Sympathetic* or *reactional causes.*—There are many diseases in the course of which meningitis is developed, or which, at least, become complicated by nervous symptoms. It is very often seen to supervene upon erysipelas of the scalp or face, either by sympathy (and here let us remark in passing, that this expression serves but to mask our ignorance of the real nature and *modus operandi* of such causes) or by the inflammation being propagated to the covering of the brain through the orbital openings, or through the medium of the veins communicating between the scalp and sinuses of the dura mater. M. Foville has observed that it declares itself sympathetically in certain affections of the serous membranes; and he mentions having seen a case of hydrocele, operated for by puncture and injection, followed by inflammation of the synovial sacs, producing anchylosis of the involved articulation, and finally succeeded by all the rational signs of acute meningitis. We ourselves have had more than one example of inflammation of the membranes of the brain occurring as a coincidence of violent acute articular rheumatism. Happily, however, this coincidence is extremely rare. Sympathetic meningitis occurs very often in typhoid fever, not as an element of the latter affection, but as a complication, and it is to this variety that the name of *typhoid fever of the ataxic form* is applied. We here naturally ask whether there exists any mechanism by which it is possible for the affection of the glands of Peyer to react upon the brain and its coverings. We feel unwilling to pronounce too positively on this subject, but of this we are convinced, that a large proportion of the cases of meningitis regarded as typhoid are really idiopathic, and entirely independent of the intestinal lesion, having either preceded, or come on simultateously with the affection of the glands of Peyer, in either case precisely as though the latter had not existed. However, we do not refuse altogether to admit that there is such a disease as real secondary meningitis, developed during the course and under the influence of typhoid fever; but instead of saying that it is produced by sympathy, by an imponderable cause, inappreciable by any of the senses, is it not more plausible and exact to say, that it is produced by poison, the typhoid element, the septic principle, which, diffused through the blood and the whole economy, acts upon the nervous centres and their appendages as matter acts upon matter? In either case it is very certain that in our service where this general infection is prevented, the development of the ataxic phenomena of typhoid meningitis is prevented also, so that, in reality, they occur in our wards only as extremely rare exceptions.

The method which we have followed in describing the symptoms of meningitis sufficiently indicates its ordinary course, and we are saved the necessity of discussing this point in a special manner; a word, however, as regards its duration.

When the disease is really idiopathic and violent, it often carries off patients, especially infants, during the first week, at latest before the expiration of the second; and sometimes, so rapid is its progress, it removes the sufferers between the first and fourth days.

Slow nervous fever is but mild sympathetic meningitis, occurring with other affections.

The prognosis is generally grave, and the physician should carefully guard against allowing himself to be deceived by the lucid moments and intervals of calm which frequently occur, but which are so often and so quickly succeeded by renewed and more alarming paroxysms.

We now hasten to an important portion of our subject, namely, the treatment.

*Treatment.*—The treatment which answers best in meningitis is the antiphlogistic. In what manner should it be employed? We are able to affirm to you that the method of copious sanguine depletions is preferable to any other. But you must not believe that you will often obtain happy results from the first bloodletting; it is generally not till after the lapse of two or three days, during which you have insisted upon repeated bleedings, that you will see the disease yield, the delirium diminish, the fever subside, etc. And yet I must tell you, that in this inflammation the most energetic treatment most opportunely applied, is far from being attended by the same pleasing results furnished in phlegmasias of other organs. I would not be far from the truth in saying, that the most judiciously prescribed and vigorously employed venesections will scarcely effect the cure of half the cases of meningitis which fall into your hands.

Of the thousand other remedies that have been proposed in this disease it is sufficient to remark, that they should not all be discarded; on the contrary, there are a few which may always be advantageously associated with sanguine emissions. Thus, to the best of antiphlogistics, that is to say, to bleeding, leeches, diluent drinks, cold drinks, ice employed internally and as a topical remedy; and to revulsives, those powerful adjuvants to the antiphlogistic treatment properly so called, that is to say, blisters, (camphorated in preference) applied to the inferior extremities or even to the scalp itself, which should be previously shaved,—to all these means, we say, you should add musk, camphor, castor, etc., which, incontestably, in a great number of cases, have produced favorable results. As to opium, or tartrate antimony, or sulphate of quinine, all of which have been used in meningitis, without dwelling here upon their inefficiency, or inconveniences, we will only say that in despair of the case, and in the absence of any good results from your other means, you may try them, provided you administer them with prudence and moderation.

In enumerating the antiphlogistic agents which you may, and which you even must use, we omitted to mention one in every respect worthy of recommendation, the effects of which in a number of cases have been, so to express ourselves, powerful. You need scarcely be told that we allude to cold affusions. They should be frequent, repeated, and carefully and opportunely combined with the means previously enumerated. Guersant and Foville extol these *bains d'affusion* very highly, and make great use of them, but they differ as to the period of the disease when they should be employed. M. Guersant very seldom prescribes them until the accidents of the first period have given place to drowsiness and coma; and we have derived good effects under similar circumstances. M. Foville, on the contrary, thinks that they should be employed as the principal remedy from the commencement of the disease, bleeding, leeches, and other means being conjoined merely as adjuvants.

But, to close our remarks, meningitis is one of those diseases in the treatment of which you must arm yourself with courage and perseverance, and act with energy. Happy are you when you have time to bring to bear against this disease all the powerful means possessed by the art; for there are cases of meningitis (occurring in children particularly) that may be called frightful: that in the space of twenty-four or thirty-six hours carry off the patients before you have had time to do more than choose your weapons.

At a late meeting of the Academy of Sciences, Dr. Lesauvage presented a memoir entitled "*Physical considerations on Small-pox and its Treatment.*" The author insists principally upon the necessity of resorting in the first moments of the disease to the antiphlogistic method. He reported many cases in which the application of this plan had been followed by complete success while in the same hospital and during the same epidemic, other patients with whom sanguine emissions were not practised died of acute cerebritis. M. Lesauvage is far from considering cauterization of the pustules unimportant; but he believes that this practice, the principal result of which is to prevent the disease from leaving its traces, and which contributes at the same time to moderate the secondary fever, would be, in the majority of cases, insufficient to induce a happy termination. He thinks, moreover, that most frequently the antiphlogistic treatment when employed in time and without timidity, is adequate to prevent the deforming cicatrices which are so often left on the face by variola treated according to the ancient method.

I have more than once alluded to the fact that Dr. Brooks, a young physician from Boston, is here preparing himself for teaching in the United States Veterinary Surgery. He was in Paris prosecuting the study of medicine when he heard, that the Agricultural Society of Massachusetts had offered four hundred dollars, *per annum*, for two years, to any one who would spend that time in the Veterinary school at Alfort, preparatory to teaching veterinary medicine in Boston. With a commendable zeal he offered himself for the undertaking, and now for twelve months has been most assiduously engaged fitting himself for the duty. The result of this effort, it is hoped, may be the establishment, by the Commonwealth of Massachusetts, of a school of veterinary medicine. The Agricultural Society has been fortunate in securing the services of an individual of the zeal manifested by Dr. Brooks in the enterprise, and who at the same time is possessed of so much talent for carrying it into effect. With my next letter I shall have the pleasure of presenting you with an interesting communication from Dr. B. on the subject.

As medical reform is a subject much talked of in our country, I will report something in my next concerning the medical reform bill which is soon to come up in the French House of Peers. For the present I will not trespass longer upon the patience of your readers.

Paris, May 30th, 1847.

